# Cannabinoid Receptors Modulate Physiological Remodelling of the Blood–Testis Barrier

**DOI:** 10.1002/jcp.70109

**Published:** 2025-11-18

**Authors:** Francesco Manfrevola, Giulia Ricci, Antonio Suglia, Vincenza Grazia Mele, Antonella Migliaccio, Rosanna Chianese, Gilda Cobellis, Teresa Chioccarelli

**Affiliations:** ^1^ Department of Experimental Medicine University of Campania “Luigi Vanvitelli” Naples Italy; ^2^ Univ Rennes, Inserm, EHESP, Irset (Institut de recherche en santé, environnement et travail) – UMR_S 1085 Rennes France

**Keywords:** blood–testis barrier, CB1 and CB2 receptors, Occludin, spermatogenesis

## Abstract

The endocannabinoid system, including the CB1 and CB2 receptors, has been associated with the modulation of blood–brain barrier and gut barrier. Herein, using CB1 knock‐out male mice, we studied the potential role of these receptors in maintenance of blood–testis barrier (BTB) integrity during the seminiferous epithelium remodelling phase (Stages VIII–XI), focusing on events responsive to CB1 and CB2 activity. Our results showed that the genetic loss of CB1 disrupted testicular expression of some components of BTB, including factors of junctional complexes, promoting tubular infiltration of blood cells. Such infiltration specifically occurred at Stages VIII–IX transition. Gene expression analysis of molecular tags that highlight BTB remodelling (by addressing Occludin to early/late endosome, membrane recycling and proteasome) revealed higher BTB dynamism and impoverishment of tight junctions at Sertoli–Sertoli interface with significant effects on BTB remodelling activities. In detail, CB1 deletion increased kinetic of internalization and recycling of tight junctions and simultaneously promoted proteosome‐mediated Occludin degradation with negative effects on permeability of BTB during its remodelling. This caused the leakage of the tight junctions, the premature passage of germ cells in adluminal compartment and downstream the slowing of spermatogenesis. These results strongly indicated that CB1 and CB2 activation contribute to BTB remodelling being both involved in the modulation of tight junction‐associated proteins and in their dynamism: these data highlight a new role for CB1 in spermatogenesis.

## 1 Introduction

In mammals, male germ cell progression occurs in the seminiferous epithelium tubular compartment where somatic Sertoli cells organize a number of spermatogenetic stages (numbered I–XII in mouse testis), sequentially, spatially and temporally arranged along the longitudinal axis of the tubule. Such stages represent a unique association of developing germ cells in the different layers of the seminiferous epithelium (Hess and De Franca 2008). Sertoli cells control cell progression by cell–cell communications and junctions (Sharpe 1986). In particular, adjacent Sertoli cells organize, at base‐lateral level, the blood–testis barrier (BTB) that is a complex of different types of cell junctions, as tight junctions (TJs), gap junctions (GJs), desmosome‐like junctions and the basal ectoplasmic specializations (i.e., atypical adherent junctions, AJs) (Lui and Cheng 2007). The BTB physically divides the seminiferous epithelium in two compartments structurally and functionally separated: (i) the basal compartment, in contact with the systemic circulation, that includes spermatogonia (SPGs) and developing preleptotene spermatocytes (SPCs); (ii) the luminal compartment, immunologically isolated from systemic circulation, that contains meiotic SPCs and post‐meiotic spermatids (SPTs) (Cheng and Mruk 2012). However, unlike other blood–tissue barriers, the BTB is a highly dynamic structure (Yan and Cheng 2005; Chihara et al. 2010) since, at Stages VIII–IX of the epithelial cycle in the mouse, TJs and AJs need to be transiently remodelled to guarantee the passage of preleptotene SPCs in the luminal compartment. This event occurs thanks to vesicle trafficking of endocytosed inter‐Sertoli junctional proteins that dynamically ensures the timely opening–closing of the BTB without affecting barrier integrity and permeability. Indeed, mechanisms of endocytosis and recycling (i.e., transcytosis) promote alterned disassembly and reassembly of inter‐Sertoli cell junctions. The coexistence of TJs and AJs, each other engaged via peripheral adaptors such as the zonula occludens‐1 (ZO‐1) protein, fortifies the BTB integrity, particularly while it remodels. Notably, when AJ is restructuring, the TJ transiently replaces its function and vice versa. This rapid dynamism, namely BTB remodelling or dynamic, promotes transient opening of BTB and the organization of new junctional complexes at the basal level, just below the preleptotene SPC in transit (Chihara et al. 2010; Wen et al. 2016; Wen, Tang, Gao, et al. 2018; Wen, Tang, Li, et al. 2018). Recently, ubiquitination has been associated with endocytic removal, trafficking and sorting of transmembrane proteins, including proteins of cell junctions, revealing an ubiquitin‐based protein trafficking mechanism (Lui and Cheng 2007). The ubiquitinated proteins may either be degraded via proteasome or deubiquitinated and recycled at the plasma membrane to accommodate junction's reassembly (Lui and Cheng 2007).

Several signals and factors have been described to regulate, or participate to modulation of BTB activities, particularly testosterone and cytokines (Lui and Cheng 2007; Su et al. 2010; Catizone et al. 2012; Ghafouri‐Fard et al. 2021). Both factors regulate BTB remodelling via differential effects on kinetic equilibrium between endocytosis and transcytosis by addressing endocytosed proteins to membrane recycling or lysosome, respectively (Yan et al. 2008).

In addition, also other non‐steroidal metabolites play a role in the maintenance of the BTB; among these, the arachidonic acid, an essential fatty acid, is important for the BTB modulation since it can damage the TJ integrity under hyperthermic conditions (Hu et al. 2023). Interestingly, free arachidonic acid is functionally interlinked with different lipid signalling networks including those involving prostanoid pathways and the endocannabinoid system (ECS) (Rouzer and Marnett 2011). In this regard, endocannabinoids, in addition to being ligands of CB receptors, are a source of arachidonic acid that can then be a substrate for the production of prostaglandins, with potential effects on BTB regulation.

Noteworthy, the endocannabinoids, anandamide (AEA) and 2‐arachidonoylglycerol (2‐AG), as well as their receptors, the cannabinoid receptors CB1 and CB2, have been associated with modulation of blood–brain barrier and gut barrier (Yang et al. 2016; Alhamoruni et al. 2010; Lu et al. 2008; Hind et al. 2015; Vendel and de Lange 2014). No data are available on their potential role in the BTB regulation. Noteworthy, it is currently accepted that cannabinoid consumption leads to male endocrine physiology alteration and hypothalamus–pituitary–gonad axis dysfunction (Maccarrone et al. 2021; Fonseca and Rebelo 2022).

In both mouse and rat, CB1 has been localized in Leydig cells and SPTs. This receptor has been related to steroidogenesis and differentiation of Leydig cells (Meccariello et al. 2014; Wenger et al. 2001; Cobellis et al. 2016; Pierantoni et al. 2009; Cacciola et al. 2008), as well as to chromatin remodelling events associated with histone displacement in SPTs and protamine thiol oxidation in spermatozoa (SPZs) (Cacciola, Chioccarelli, Fasano, et al. 2013; Chioccarelli et al. 2010; Chioccarelli, Manfrevola, et al. 2020; Chioccarelli, Pierantoni, et al. 2020). Interestingly, in rat testis, a weak and stage‐specific expression of CB1 has been observed in Sertoli cells at Stages VII–X (Pierantoni et al. 2009; Cacciola et al. 2008). To date, there is a gap of knowledge about the role of CB1 in Sertoli cell physiology and function.

To fill‐in this gap of knowledge, herein we investigated the effects of CB1 gene deletion on the expression of genes coding for BTB organizing proteins. Therefore, focusing on Occludin (OCLN) as the main protein of BTB TJs. We further investigated the role of CB1 and CB2 in TJ trafficking‐based BTB remodelling.

## 2 Materials and Methods

### 2.1 Experimental Animals

Mice (*Mus musculus*) genetically deleted for CB1 were provided by Prof. Ledent (Ledent et al. 1999). Male and female CB1 heterozygous (CB1^+/−^) mice have been maintained on a CD1 background (Charles River Laboratories, Lecco, Italy) to expand the colony, then used to generate adult wild‐type (WT), CB1^+/−^ and CB1^−/−^ male mice. All the animals were kept in a room with controlled temperature (22°C ± 2°C), ventilation and lighting (12‐h light/dark cycles) and were maintained on a standard pellet diet with free access to water. The genotype was determined by PCR analysis of genomic DNA (gDNA) using specific primers for the neomycin cassette (mutated allele) and *Cb1* gene as previously reported (Cuomo et al. 2022).

The number of enroled adult animals was determined by the parameters that we have to adopted for the G*Power analysis required to get the permission for in vivo experiments in Italy, suggested by the Legal Entity giving the permission.

Experiments were approved by the Italian Ministry of Education and the Italian Ministry of Health, with authorization n° 48/2022‐PR issued on 28.01.2022. Procedures involving animal care were carried out in accordance with the National Research Council's publication *Guide for Care and Use of Laboratory Animals* (National of Institutes of Health Guide).

### 2.2 In Vitro Testes Incubation

Arachidonyl‐2′‐chloroethylamide (ACEA) and 3‐(1,1‐dimethylbutyl)‐6aR,7,10,10aR‐tetrahydro‐6,6,9‐trimethyl‐6H‐dibenzo[b,d]pyran (JWH‐133), two selective agonists for CB1 and CB2, respectively, were obtained from Cayman Chemical (Michigan, USA). The drugs were of the purest analytical grade and each was dissolved in dimethylsulfoxide (DMSO), according to the manufacturer's instructions.

All the experiments were carried out on CB1^+/−^ adult testes (*n* = 9/experimental group). Thus, testes were rapidly removed, washed, engraved with a feeble notch in *tunica albuginea* and finally, incubated in PBS for 90 min at RT.

To preserve control quality and avoid any effect due to the vehicle, all experimental groups received DMSO (0.05%), being ACEA and JWH‐133 dissolved in DMSO.

The control group was treated with vehicle, while the other groups with ACEA or JWH‐133 at three different concentrations (0.1–1–10 µM). After treatments, some testes (*n* = 5) were stored at −80°C and then used for total RNA or protein preparation while others (*n* = 4) were used to the FITC‐dextran diffusion assay as described below.

### 2.3 Total RNA Preparation, cDNA Synthesis and *RTqPCR* Analysis

Total RNA was isolated from WT and CB1^−/−^ testes (*n* = 5/genotype, at least) or CB1^+/−^ testes in vitro treated (*n* = 5 for experimental group) as described before, using TRIZOL Reagent (Invitrogen Life Technologies, Paisley, UK) according to the manufacturer's instructions. To remove potential contamination of gDNA, RNA aliquots (10 μg) were treated with 2 U DNase (RNase‐free DNase I, Ambion, Thermo Fisher Scientific, MA, USA) according to the manufacturer's instructions. Purity and integrity of RNA samples were routinely determined.

The cDNA synthesis was carried out in 20 µL final volume containing 1× first strand buffer, 5 µg of RNA, 0.5 µg oligo dT_(18)_, 0.5 mM dNTP mix, 5 mM DTT, 40 U RNase Out (Invitrogen Life Technologies, Paisley, UK), 200 U SuperScript‐III RnaseH^−^ Reverse Transcriptase (Invitrogen Life Technologies, Paisley, UK). As a negative control, cDNA was synthesized without adding the reverse transcriptase enzyme.

The RTqPCR analysis was performed according to the manufacturer's instructions (CFX‐96; Bio‐Rad, Segrate, Italy) in a 20 µL reaction mixture (SyberGreen; Bio‐Rad, Segrate, Italy) containing diluted cDNA (1:5). Assays were performed in triplicate, and a standard curve from consecutive 5‐fold dilutions (2 µg–31 ng) of a cDNA pool representative of all the samples was included for PCR efficiency determination. The gene expression analysis, corrected for PCR efficiency and normalized towards the reference gene (ribosomal protein S18, *Rps18*), was performed by CFX Manager software (Bio‐Rad, Segrate, Italy). For details about genes, primer sequences, annealing temperatures and product size, see Table 1. Results from independent cDNA/genotype, each analyzed in triplicate, were expressed as mean value of normalized fold expression (nfe) ± SEM.

**Table 1:** Primer sequences (S: sense; AS: antisense) and annealing temperature (*T*_m_) for RTqPCR analysis.

Gene primers	Sequences 5′–3′	*T*_m_ (°C)	Product size (bp)
*Zo‐1* S *Zo‐1* AS	gcaccatgcctaaagctgtc actcaacacaccaccattgc	57	122
*Cldn‐5* S *Cldn‐5* AS	agagcagaggcaccagaatc acacagcaccagacccagaa	57	143
*Ocln* S *Ocln* AS	cctactcctccaatggcaaa ctcttgccctttcctgcttt	55	208
*Eea1* S *Eea1* AS	ctgacacccacacaactgtcc tgaaggctggaaagcaaact	58	189
*Rab13* S *Rab13* AS	tggatgaggctttcagttcc agtccgactccctcaggtct	58	197
*Rab9* S *Rab9* AS	tctgactgttgcctgcttacat gcttgggcttcttctgtagaca	56	182
*Itch* S *Itch* AS	gccatacttgttttaaccgcc gatgtgcaacaggcagagaa	56	189
*Rps18* S *Rps18* AS	gagactctggatgctaactag ggacatctaagggcatcacag	56	172

### 2.4 Protein Extraction and Western Blot Analysis

WT and CB1^−/−^ testes (*n* = 5/genotype, at least) or CB1^+/−^ testes in vitro treated (*n* = 5 for experimental group) as described before were sonicated in RIPA buffer [PBS, pH 7.4, 10 mM dithiothreitol, 0.02% sodium azide, 0.1% SDS, 1% Nonidet P‐40, 0.5% sodium deoxycholate, in the presence of protease inhibitors (10 μg/mL of leupeptin, aprotinin, pepstatin A, chymostatin and 5 μg/mL of TPCK)] and analyzed by western blot. Briefly, proteins were separated by SDS‐PAGE (10% acrylamide) and transferred to polyvinylidene difluoride membrane (GE Healthcare, Milano, Italy) at 280 mA for 2.5 h at 4°C. Filters were treated for 3 h with blocking solution [5% non‐fat milk, 0.25% Tween‐20 in Tris‐buffered saline (TBS, pH 7.6)] and then incubated overnight, at 4°C in TBS–milk buffer (TBS pH 7.6, 3% non‐fat milk) with different primary antibody. After washing in 0.25% Tween20–TBS, filters were incubated with the secondary antibodies, diluted 1:1000 in TBS–milk buffer and then washed again. The immune complexes were detected using the enhanced chemiluminescence‐western blotting detection system (Merk Life Science, Milano, Italy). Antibodies and relative dilutions are reported in Table 2.

**Table 2:** Primary antibodies used for western blot and IF analyses.

Primary antibody	Dilution	Provider (Catalogue Nr.)
Vimentin	1:2000 (WB)	Elabscience (E‐AB‐27405), Houston, USA
β‐catenin	1:1000 (WB)	Santa Cruz (sc‐7199), Texas, USA
β‐actin	1:2000 (WB)	Elabscience (E‐AB‐20031), Houston, USA
F‐actin	1:1000 (WB)	Thermo Fisher (MA1‐80729), MA, USA
Occludin	1:500 (WB) 1:100 (IF)	Thermo Fisher (40‐4700), MA, USA
ZO‐1	1:500	Santa Cruz (sc‐33725), Texas, USA
ERK‐2	1:1000	Santa Cruz (sc‐1647), Texas, USA
CB1	1:1000	Santa Cruz (sc‐293419), Texas, USA

Signals were quantified by densitometry analysis, adjusted relatively to ERK1/2 levels and graphed as OD fold change (mean ± SEM). The specificity of immunoreaction has already been demonstrated (Cobellis et al. 2010; Cacciola, Chioccarelli, Altucci, et al. 2013; Acone et al. 2009; Trabucco et al. 2009), and here routinely checked by omitting primary antibody (data not shown).

### 2.5 Spermatogenetic Stage and Tubular Infiltration Analysis

Testes from WT and CB1^−/−^ mice (*n* = 5/genotype, at least) were dissected, fixed in Bouin's solution and processed for histological examination using standard procedures. Gonads were embedded in paraffin, sectioned with a microtome (three serial sections, 7 μm each, from apical, central and caudal testicular regions), stained with hematoxylin–eosin staining (H&E) and observed under a light microscope (Leica Microsystems Inc., Milano, Italy). Images were captured using a high‐resolution digital camera (DC300F; Leica Microsystems Inc., Milan, Italy) and, in combination with the direct observation at microscope, these were used to study the incidence of: (i) the spermatogenetic stages relative to early meiotic phase of Prophase I (i.e., Stages VIII up to XI) and (ii) white blood cell infiltration in tubular compartment (i.e., tubular infiltration).

Serial sections were specifically used to properly identify the spermatogenetic stages. The identification of Stages VIII–XI was based on specific features of SPCs and SPTs as previously reported (Oakberg 1956). In particular, tubules at Stage VIII were identified by the presence of SPZ lining the luminal surface of the seminiferous epithelium with tails addressed to the lumen. The identification of tubules at Stage IX and grouped Stages X–XI was based on big size of pachytene SPCs and specifically discriminated by nuclear shape of SPTs and relative chromatin condensation state (Steps 9 and 10–11 of spermiogenesis, respectively) (Catizone et al. 2012; Oakberg 1956).

Testicular sections from WT and CB1^−/−^ mice (*n* = 5/genotype) were used to identify and count tubules at Stages VIII (containing preleptotene SPCs), IX (containing leptotene SPCs) and X–XI (containing leptotene to zygotene SPCs), as well as to count total tubules. A total of 610 tubules from 5 different WT testes and 750 tubules from 5 different CB1^−/−^ testes were examined and used to calculate the percentage of tubules at Stages VIII, IX and X–XI.

Testicular sections from WT and CB1^−/−^ mice (*n* = 5/genotype) were used to count total and infiltrated tubules. The percentage of infiltrated tubules was calculated relatively to total tubules.

Sections from WT and CB1^−/−^ testes (*n* = 5/genotype at least) were used to identify and count the spermatogenetic stages of the infiltrated tubules (i.e., infiltration window). Because of a stage‐specific infiltration, the percentages of tubules specifically infiltrated at Stages VIII, IX and X–XI were calculated relatively to the infiltration window in order to express data independently by germ cells progression delay.

All the counts were validated using double‐blind testing by two observers.

### 2.6 Immunofluorescence Analysis

Testis from WT and CB1^−/−^ mice (*n* = 3/genotype, at least) or Control (CB1^+/−^) and CB1^+/−^ ACEA and JWH‐133 in vitro treated testes (*n* = 3 for experimental group) as described before were fixed in 4% paraformaldehyde (sc‐281692; Santa Cruz Biotechnology, Heidelberg, Germany) and processed for immunofluorescence analysis using standard procedures.

Testis sections were permeabilized with PBS pH 7.4 containing 0.1% Triton‐X‐100 (X100; Sigma‐Aldrich, Milano, Italy) for 30 min. After, blocking was conducted with 10% of donkey serum (ab7475; Abcam, Cambridge, UK) for 30 min at RT and the sections were then incubated overnight at 4°C with anti‐OCLN antibody (for details, see Table 2). Following three washes in Dulbecco's PBS (DPBS, 1X), a fluorescein isothiocyanate (FITC) (excitation 460–500 nm; emission 512–542 nm) conjugated Ab (711‐095‐152; Jackson ImmunoResearch, Cambridge, UK) was used for 1 h at 37°C. Nuclei were labelled with DAPI (D9542; Sigma‐Aldrich, Milano, Italy) (excitation 340–380 nm; emission 435–485 nm) and the slides were observed (Leica DM5000 B + CTR 5000; Leica Microsystems, Wetzlar, Germany) with a UV lamp. The images were captured using a 40× objective and acquired using IM 1000 software (version 4.7.0) and a Leica DFC320 R2 digital camera. The green fluorescence signal was quantified using the Nikon Imaging Analytical Software (NIS‐Elements Analysis D 4.40.00, 64 bit).

### 2.7 BTB Permeability Test by FITC‐Dextran Diffusion Assay

To check the BTB occluding junction functionality, Control (CB1^+/+^ and CB1^+/−^) and CB1^+/−^ ACEA and JWH‐133 treated testes were subjected to FITC‐dextran diffusion assay (Devraj et al. 2018). Briefly, *tunica albuginea* of control and treated testes was gently removed, and testes were immersed for 2 h at body temperature (34°C) in a solution of 1 mg/mL FITC dextran 4 kDa (IKF801 Immunological Sciences, Italy) in RPMI 1640 supplemented with 10% foetal bovine serum and penicillin/streptomycin. After this incubation, FITC‐dextran was removed, and testes were embedded in OCT Tissue freezing medium and frozen in liquid nitrogen. Then, samples were sectioned by cryostat (section thickness 6 µm), observed and photographed by a Fluorescence Microscope (Nikon Eclipse, Nikon, Japan). The green fluorescence signal diffused in the luminal compartment was quantified using the Nikon Imaging Analytical Software (NIS‐Elements Analysis D 4.40.00, 64 bit) (see Figure S3). To determine the localization of FITC‐dextran tracer, the fluorescent signal was superimposed with phase contrast images (see Figure S4).

### 2.8 Statistical Analysis

ANOVA followed by Duncan's test (for multigroup comparison) or Student's *t*‐test (for two independent group comparison) was conducted to evaluate the significance of differences. Data were expressed as the mean ± SEM.

## 3 Results

### 3.1 Impact of CB1 Gene Deletion on Expression of BTB‐Related Proteins and BTB Integrity

**Figure 1:**
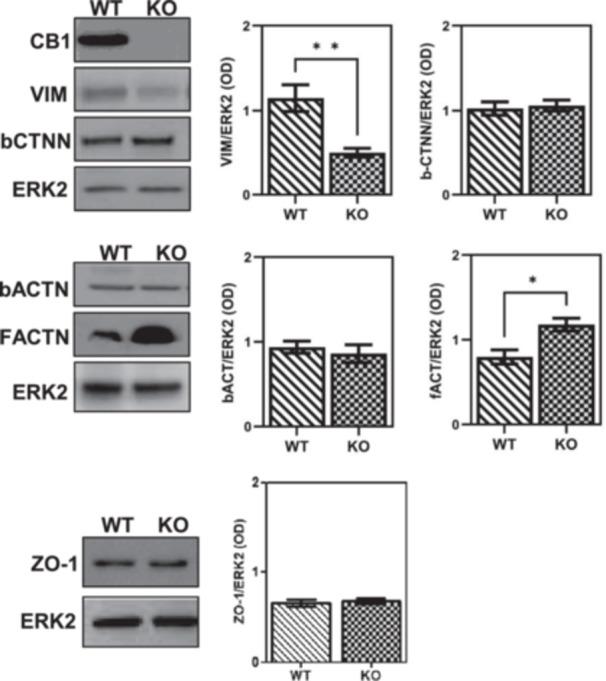
Gene expression analysis of CB1 and BTB components in WT and CB1^−/−^ (KO) testes. Western blot analysis of CB1, Vimentin (VIM), β‐catenin (β‐CTNN), β‐actin (β‐ACTN), F‐actin (F‐ACTN) and ZO‐1. Protein amounts were quantified by densitometry analysis, normalized against ERK1/2 signals and expressed in OD. All the data were reported as mean value ± S.E.M. (*p < 0.05; **p < 0.01).

We studied the role of CB1 in maintenance of BTB integrity during the remodelling phase using WT and CB1^−/−^ testes. First, the absence of CB1 protein in CB1^−/−^ testis was monitored and further verified by western blot analysis (Figure 1). Then, we focused on events responsive to 2‐AG/CB2 activity and mitotic–meiotic switch of germ cells. Gene expression of TJ components [i.e., zonula occludens protein‐1 (*ZO‐1*) and F‐actin (F‐ACTN)], as well as of adaptors [β‐catenin (β‐CTNN)] and scaffolding [β‐actin (β‐ACTN), Vimentin (VIM)] proteins related to BTB junctions was evaluated in WT and CB1^−/−^ testis using western blot analysis (Figure 1).

Western blot analyses revealed a lower expression of VIM (*p* < 0.01) and a higher expression of F‐ACTN proteins in CB1^−/−^ testis compared to WT. No change was observed when we analyzed the expression of β‐CTNN and β‐ACTN proteins (Figure 1). These results suggested that CB1 deletion targeted the availability of components of cytoskeleton scaffold associated with inter‐Sertoli cell junctions.

**Figure 2:**
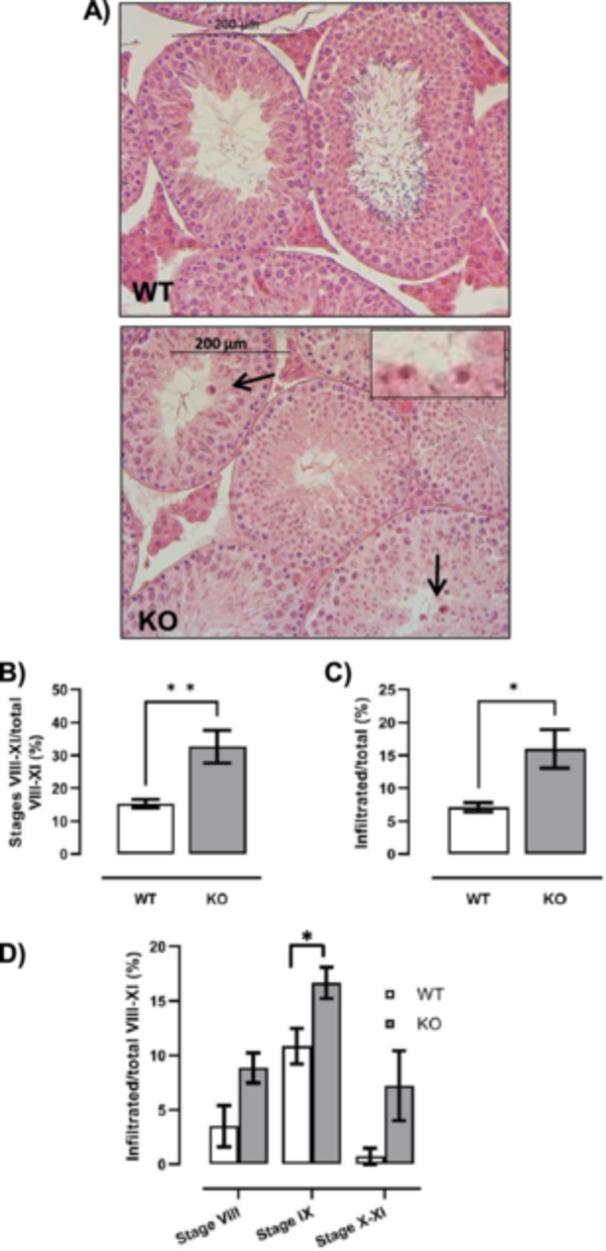
Histological analysis of seminiferous tubules from WT and CB1^−/−^ (KO) testes. H&E staining of WT and CB1^−/−^ (KO) testes: inset shows infiltrated blood cells (scale bar: 200 μm; inset scale bar: 10 μm) (A). Number of Stages VIII, IX and X–XI relatively to total tubules (B). Number of infiltrated tubules relatively to total tubules (C). Infiltrated tubules at Stages VIII, IX and X–XI relatively to the infiltration window (tubules at Stages VIII–XI) (D). All the data were reported as mean values ± S.E.M. (*p < 0.05; **p < 0.01).

Additionally, to better characterize the BTB remodelling phase occurring when preleptotene SPCs migrate from basal to adluminal compartment, H&E‐stained sections from WT and CB1^−/−^ testes were analyzed to study germ cell progression during early phases of meiosis (Figure 2A). In particular, testicular sections were analyzed at the histological level and studied by classifying and counting tubular stages (Figure 2B) within the proper window of the spermatogenetic cycle (Stages VIII–XI).

The histological analysis of tissues revealed the presence of white blood cell infiltration in the tubular compartment (Figure 2A). This occurred independently by genotype, either WT or CB1^−/−^, although we counted a few infiltrated cells (data not shown). However, the number of infiltrated tubules was higher in CB1^−/−^ than in WT (*p* < 0.05) (Figure 2C). The infiltrated cells appeared specifically at Stages VIII–XI (i.e., infiltration window). Relatively to the infiltration window, the percentage of tubules at Stages VIII–XI was higher in CB1^−/−^ than WT (Figure 2B) while the relative distribution of the stages showed that the percentage of infiltrated tubules at Stage IX was significantly higher (*p* < 0.05) in CB1^−/−^ than WT testis. Number of infiltrated tubules at Stages VIII or X–XI did not change in WT and CB1^−/−^ mice (Figure 2D), indicating that CB1 deletion impaired BTB functional integrity, specifically during transition from Stages VIII–IX.

### 3.2 Impact of CB1 Gene Deletion on Junctional Protein OCLN and Dynamic Remodelling of BTB

**Figure 3:**
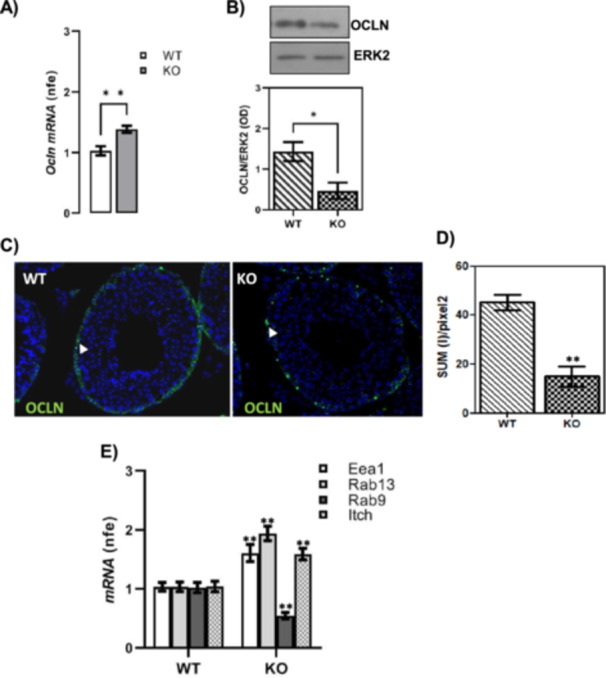
Gene expression analysis of OCLN and markers related to its trafficking in WT and CB1^−/−^ (KO) testes. RTqPCR analysis of Ocln mRNA. Transcript amounts were reported as normalized fold expression (nfe) relatively to Rps18 gene (A). Western blot analysis of OCLN. Protein amounts were quantified by densitometry analysis, normalized against ERK1/2 signals and expressed in OD values (B). Immunofluorescence analysis of OCLN; white arrowheads represent OCLN localization (green) in testis. Nuclei were labelled with DAPI (blue). Scale bar: 5 µm (C). Quantitative analysis of OCLN green fluorescence on supra‐basal epithelium Regions of Interest (ROI); values are expressed as Sum of Intensity/area (SUM (I)/pixel^2^), measured using the Nikon Imaging Analytical Software (NIS‐Elements Analysis D 4.40.00, 64 bit) (D). RTqPCR analysis of endocytic (Eea1), recycling (Rab13) and degradation (Rab9 and Itch) markers of OCLN. Transcript amounts were reported as normalized fold expression (nfe) relatively to Rps18 gene (E). All data were reported as mean value ± S.E.M. (*p < 0.05; **p < 0.01).

Gene expression analysis of the Occludin (OCLN), the main protein of TJs, was carried out using RTqPCR, western blot and immunofluorescence analyses (Figure 3A–D).

The expression levels of *Ocln* mRNA (*p* < 0.01) were higher in CB1^−/−^ than WT testis (Figure 3A) whereas the protein levels were significantly downregulated in CB1^−/−^ testis compared to WT (*p* < 0.05) (Figure 3B). Accordingly to the latter result, OCLN immunofluorescence analysis revealed a more intense OCLN signal in WT compared to CB1^−/−^ testis sections (Figure 3C–E), indicating a partial loss of this protein to the inter‐Sertoli BTB junctions. In particular, in WT testis, OCLN appears correctly localized at the basal compartment of seminiferous tubules with a wavy pattern at the basolateral membrane region of adjacent Sertoli cells, and it is completely absent in higher levels of the epithelium (Figure 3C). Conversely, in CB1^−/−^ testis, an irregular distribution pattern of OCLN was observed at the periphery of the tubules (Figure 3D). Therefore, we can conclude that CB1 gene deletion affected OCLN protein availability and distribution pattern, even if the increase of OCLN mRNA suggested the potential trigger of restoring mechanism of TJ integrity. According to this hypothesis, we observed that even ZO‐1 and Claudin‐5 mRNAs (*Zo‐1* and *Cldn‐5*) significantly increase in CB1^−/−^ testis with respect to WT (Figure S1). Taken together, these results indicate a possible alteration of TJ recycling in CB1^−/−^ testes. To address this point, we examined gene expression of *Eea1* and *Rab9*, the early and late endosome formation markers, respectively (Yan and Cheng 2005; Yan et al. 2008). In addition, we also studied gene expression of *Rab13* and *Itch*, specifically related to endocytic recycling (Marzesco et al. 2002; Morimoto et al. 2005) and ubiquitin‐mediated degradation (Traweger et al. 2002; Lui and Lee 2005) of the OCLN protein, respectively. RTqPCR analysis revealed that *Eea1*, *Rab13* and *Itch* transcripts were significantly up regulated (*p* < 0.01) in CB1^−/−^ testes compared to WT while *Rab9* mRNA expression decreased in CB1^−/−^ versus WT (*p* < 0.01) (Figure 3F), suggesting that CB1 absence altered endocytosis and recycling balance of TJs.

### 3.3 CB1 and CB2 Action on Testicular OCLN Expression

Testes from mice null of CB1 gene in heterozygous conditions (CB1^+/−^) were used as model tissue of low levels of CB1 to discriminate the responsiveness of expression of OCLN protein to CB1 and CB2 activity.

In this regard, western blot analysis on CB1^+/−^ testes revealed lower levels of OCLN (*p* < 0.01) as compared to WT animals (Figure S2A). Accordingly, the immunofluorescence analysis showed OCLN in seminiferous epithelium of CB1^+/−^, with a weaker signal in comparison to WT and with an irregular distribution pattern at the periphery of the tubules, similarly to that observed in CB1^−/−^ testis (Figure S2B,C).

**Figure 4:**
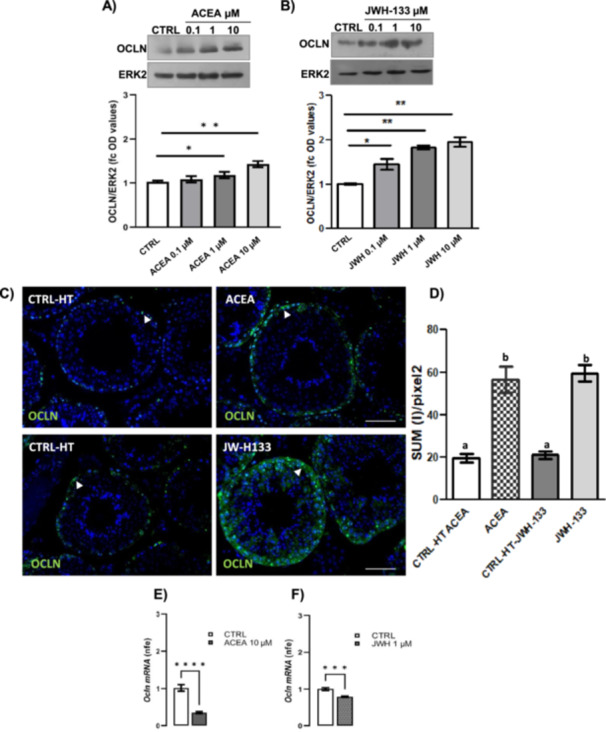
Responsiveness of OCLN expression to CB1 and CB2 receptors. Western blot analysis of OCLN in testis of HT mice in vitro treated with vehicle (CTRL), ACEA (A) or JWH‐133 (B) at different concentrations: 0.1–1–10 µM. Protein amounts were quantified by densitometry analysis, normalized against ERK1/2 signals and expressed in OD as fold change. Immunofluorescence analysis of OCLN in testis of CTRL and ACEA (10 µM) or JWH‐133 (1 µM); white arrowheads represent OCLN localization (green) in testis. Nuclei were labelled with DAPI (blue). Scale bar: 5 µm (C). Quantitative analysis of OCLN green fluorescence on supra‐basal epithelium Regions of Interest (ROI); values are expressed as Sum of Intensity/area (SUM (I)/pixel^2^), measured using the Nikon Imaging Analytical Software (NIS‐Elements Analysis D 4.40.00, 64 bit) (D). RTqPCR analysis of Ocln mRNA in testis of CTRL and ACEA 10 µM (E) or JWH‐133 1 µM (F). Transcript amounts were reported as normalized fold expression (nfe) relatively to Rps18 gene. All the data were reported as mean value ± S.E.M. (*p < 0.05; **p < 0.01; ***p < 0.001; ****p < 0.0001; a vs. b: *p* < 0.05).

With these assumptions, we carried out dose–response curves with selective CB1 and CB2 agonists (ACEA or JWH‐133, respectively). Specifically, using in vitro testis incubation system, we stimulated CB1^+/−^ testes with increasing doses of ACEA or JWH‐133 (0.1, 1 and 10 µM) and we analyzed the expression of OCLN by western blot. Densitometry analysis of signals showed that OCLN levels significantly increased after treatment with both ACEA at doses of 1 and 10 µM (Figure 4A) and JWH‐133 (Figure 4B) at all doses, demonstrating a CB1 and CB2 activity on OCLN availability and/or stability. On the basis of these results, we chose the concentration of ACEA (10 µM) and JWH‐133 (1 µM) selected as the most effective with respect to the control condition. According to western blot analyses, immunofluorescence experiment of control and treated testis revealed that OCLN availability increases in ACEA and JWH‐133 treated samples (Figure 4C,D). Notably, in the same conditions, gene expression analysis by RTqPCR revealed a compensatory increase of OCLN mRNA levels in testis treated with ACEA and JWH‐133 (Figure 4E,F).

### 3.4 CB1 and CB2 Action on BTB Function

**Figure 5:**
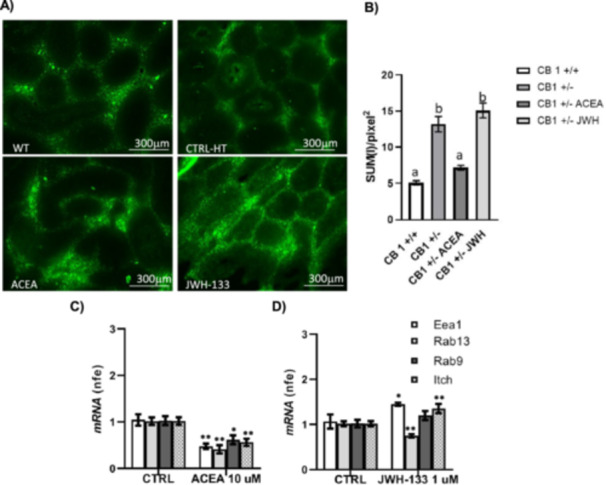
CB1 and CB2 action on BTB permeability and functionality. FITC‐dextran diffusion assay in testis of WT and HT mice in vitro treated with vehicle (CTRL), ACEA 10 µM and JWH‐133 1 µM (A) Scale bar: 300 μm. The green fluorescence signal diffused in the luminal compartment was quantified using the Nikon Imaging Analytical Software (NIS‐Elements Analysis D 4.40.00, 64 bit) as SUM (I)/pixel^2^ (see also Figure S3 for technical details) (B) a versus b: *p* < 0.05. RTqPCR analysis of endocytic (Eea1), recycling (Rab13) and degradation (Rab9 and Itch) markers of OCLN in the testis of CTRL and ACEA 10 µM (C) or JWH‐133 1 µM (D). Transcript amounts were reported as normalized fold expression (nfe) relatively to Rps18 gene. All data were reported as mean value ± S.E.M. (*p < 0.05; **p < 0.01).

To clarify the action of CB1 and CB2 on BTB function, we performed FITC‐dextran diffusion assay in CTRL and ACEA and JWH‐133 treated testes. This assay allows to study the permeability of TJs: indeed, FITC‐dextran tracer diffuses in the intercellular space, but cannot enter in the cells, therefore in the case of seminiferous epithelium, the presence of FITC‐dextran signal in the adluminal compartment indicates the leakage of BTB‐associated TJs. We observed that, as expected, FITC signal in the adluminal compartment is higher in CB1^+/−^ seminiferous epithelium with respect to WT one (Figure 5A,B). The same experimental group revealed that the treatment with ACEA of CB1^+/−^ testis restored the BTB functionality, whereas JWH‐133 did not. These results indicate the direct role of CB1 in the stabilization at the membrane level of OCLN and therefore on BTB functionality.

To better understand the different effects of ACEA and JWH in BTB functionality, we studied the already mentioned and endocytic/recycling markers (*Eea1, Rab13, Rab9* and *Itch*) by RTqPCR analysis.

Interestingly, we found that ACEA treatment caused a significant decrease of all the genes studied indicating an impairment of OCLN recycling and its stabilization at the membrane (Figure 5C). Conversely, JWH‐133 treatment leaves to the increase of *Eea1* and *Itch* indicating the activation of TJ recycling mechanism (Figure 5D). These results are in line with the previously reported OCLN immunofluorescence data (Figure 4C): even if both JWH‐133 and ACEA caused the increase of OCLN levels, the distribution pattern of this protein is significantly different in the two treatments. In ACEA‐treated samples, OCLN appeared restricted to the basal part of the epithelium, whereas in JWH‐133‐treated samples, this protein is also diffused in the cytoplasm and is observable even in the adluminal compartment.

## 4 Discussion

Several studies have demonstrated the role of ECS in permeability modulation of tissue barriers of the mammalian body, such as gut and blood–brain barrier (Yang et al. 2016; Alhamoruni et al. 2010; Lu et al. 2008; Hind et al. 2015; Vendel and de Lange 2014). To the best of our knowledge, our present data showed, for the first time, that the ECS is involved in BTB modulation. It is fair to mention that in rat testis CB1 has been observed in Sertoli cells at Stages VII–X near the BTB (Pierantoni et al. 2009; Cacciola et al. 2008), and this indicates a specific functional window of CB1 activity in the tubular compartment during spermatogenesis.

Herein, using WT, CB1^−/−^ and CB1^+/−^ mouse testes and focusing on events responsive to CB1 and CB2 activity, we studied the role of these receptors in maintenance of BTB integrity during the seminiferous epithelium remodelling phase (Stages VIII–XI).

Even if no genotype‐dependent change of the β‐CTNN adaptor or the β‐ACTN scaffold proteins (WT vs. CB1^−/−^) was observed, the analysis of VIM and F‐ACTN proteins showed that they are regulated in opposite way in these different genetic backgrounds: in detail, VIM decreased whereas F‐ACTN increased in CB1^−/−^ testes compared to WT ones. This indicates that CB1 deletion targeted gene expression profile and availability of cytoskeletal proteins involved in BTB junctional complexes (Li et al. 2015).

To verify the role of CB1 in BTB structure and remodelling, we focused on OCLN and ZO‐1 as main proteins of TJs, finding that OCLN significantly decreased in CB1^−/−^ testes being ZO‐1 protein expression not affected. Interestingly, *Ocln*, *Zo‐1* and even *Cldn‐5* mRNA increased significantly in CB1^−/−^ testis compared to WT, indicating a compensatory mechanism to restore TJ structural integrity and possibly a higher dynamism of CB1^−/−^ TJs. This also demonstrated that CB1 deletion interfered with gene expression of OCLN synthesis and/or stability.

Temporal progression of meiotic germ cells in Prophase I requires a functional BTB development (Wen, Tang, Li, et al. 2018; Wen et al. 2016; Toyama et al. 2001; Hosoi et al. 2002). Noteworthy, the histological observation and quantification of spermatogenic tubules at Stages VIII–XI, relative to preleptotene‐to‐zygotene SPCs, revealed that CB1 gene deletion affected the BTB remodelling phase with effects on its integrity. Indeed, the histological analysis of WT and CB1^−/−^ testes demonstrated that CB1 gene deletion facilitated tubular infiltration of white blood cells in the adluminal compartment. Such infiltration specifically occurred at Stage IX, demonstrating higher BTB permeability associated with the Stages VIII–IX transition and supporting the emerging role for CB1 in maintenance of the BTB integrity during the remodelling phase. Intriguingly, in accordance with toxicity animal models revealing the importance of the BTB for reinitiating spermatogenesis from undamaged SSCs after toxin‐induced aspermatogenesis (Cheng and Mruk 2012), although the genetic loss of CB1 does not impact the size of the overall germ cell population (Porreca et al. 2025), the differentiation status of the spermatogonial stem cells or undifferentiated SPGs was affected by CB1 deletion (data not shown).

In the light of these results, we analyzed the gene expression of molecular markers that address OCLN dynamics to early endosome (Yan et al. 2008), membrane recycling (Marzesco et al. 2002; Morimoto et al. 2005), lysosome (Su et al. 2010; Yan et al. 2008) and proteasome (Traweger et al. 2002; Lui and Lee 2005). Results demonstrated that the expression of *Eea1* mRNA, an early endosome formation marker, as well as of *Rab13* mRNA, an endocytic recycling marker, specifically associated with OCLN, was significantly higher in CB1^−/−^ compared to WT testes, whereas late endosome formation marker *Rab9* mRNA decreased, suggesting that the absence of CB1 increased efficiency of endocytosis and recycling of TJs. However, the gene expression of *Itch* mRNA, an E3 ubiquitin ligase that specifically addresses the OCLN to the proteasome, was higher in the CB1^−/−^ than in WT testis, indicating a high rate of OCLN degradation. This higher ubiquitin‐mediated degradation well explained the results above described such as: (i) the observed upregulation of the TJ genes (compensatory feedback on *Zo1*, *Cldn‐5*, *Ocln* mRNA), (ii) the OCLN protein deficiency, (iii) the occurrence of tubular infiltration at Stage VIII–IX during BTB remodelling. In fact, findings reported in epithelial and endothelial cells suggest that protein internalization, via endocytosis and ubiquitination, can quickly remove junction proteins from the cell–cell interface and reduce their availability at the membrane level, with disassembling effects on the junctional complexes. However, ubiquitination may either address protein to proteasome for degradation or to deubiquitinating enzymes for membrane recycling (Lui and Cheng 2007). Anyway, in primary Sertoli cells, it has been demonstrated that the expression of *Itch* addresses the OCLN to degradation leading to a perturbation of TJ permeability barrier (Lui and Lee 2005). We concluded that CB1 gene deletion perturbed BTB remodelling phase by troubling TJs trafficking (i.e., vesicle and ubiquitin‐mediated trafficking) with effects on barrier permeability/integrity at Stages VIII–IX. In agreement, several studies have demonstrated the role of ECS in permeability modulation of many tissue barriers, for example, spinal cord, gut, blood–brain barriers (Yang et al. 2016; Alhamoruni et al. 2010; Lu et al. 2008; Hind et al. 2015; Vendel and de Lange 2014).

To study the responsiveness of OCLN expression to intra‐testicular CB1 and/or CB2 activation, we carried out in vitro experiments using CB1^+/−^ testis. This represents a low CB1 level model (with similar features to CB1^−/−^) that allows pharmacological stimulation of both CB1 and CB2. Thus, CB1^+/−^ testes were incubated with vehicle or increasing doses of CB1 and CB2 agonists (ACEA and JWH‐133, respectively). We found that OCLN protein levels significantly increased after treatment with both ACEA and JWH‐133. However, the immunofluorescence analyses revealed that the OCLN localization in ACEA‐treated samples is mainly restricted to the basal TJ compartment, whereas JWH‐133‐treated samples showed a more diffused localization of OCLN both in basal and adluminal compartments. In line with these observations, the FITC‐dextran diffusion assay revealed the capability of ACEA to restore BTB functional integrity of CB1^+/−^ whereas JWH‐133 administration, in spite of the OCLN increase, cannot rescue TJ functional properties.

Notably, we also found that *Ocln* mRNA decreased as a consequence of ACEA and JWH‐133 administration, suggesting a compensatory mechanism of CB1 and CB2 stimulation on OCLN expression and/or stability. Interestingly, the analysis on endocytosis/recycling of integral membrane proteins at cell junctions revealed that *Eea1*, *Rab13*, *Rab9* and *Itch* transcripts were significantly downregulated in ACEA‐treated samples, whereas in JWH‐133‐treated samples, we observed increased levels of *Eea1* and *Itch* and lower *Rab13* content. These results suggested that CB1 and CB2 activation in opposite way contribute to BTB remodelling being both involved in the modulation of TJ‐associated proteins and in their dynamism.

We concluded that CB1 deletion increased kinetic of internalization and recycling of TJ and simultaneously promoted proteosome‐mediated OCLN degradation with negative effects on permeability of BTB during its remodelling. This promoted the leakage of the TJs, the premature passage of germ cells in adluminal compartment and downstream the slowing of spermatogenesis.

## Author Contributions

Conceptualization: Teresa Chioccarelli, Giulia Ricci, Gilda Cobellis. Methodology: Teresa Chioccarelli, Antonio Suglia, Vincenza Grazia Mele, Antonella Migliaccio, Francesco Manfrevola. Formal analysis and investigation: Teresa Chioccarelli, Antonio Suglia, Francesco Manfrevola. Writing – original draft preparation: Gilda Cobellis, Teresa Chioccarelli. Figure preparation: Teresa Chioccarelli, Vincenza Grazia Mele, Antonella Migliaccio, Francesco Manfrevola, Giulia Ricci. Writing – review and editing: Teresa Chioccarelli, Giulia Ricci, Gilda Cobellis. Visualization: Rosanna Chianese, Giulia Ricci. Supervision: Gilda Cobellis, Rosanna Chianese, Giulia Ricci. Funding acquisition: Gilda Cobellis.

## Conflicts of Interest

The authors declare no conflicts of interest.

## Supporting information

Supplementary Figures.

## Data Availability

The data sets in this study are available from the corresponding author upon reasonable request.
