# Supplementary figures and images for: Cannabinoid Receptors Modulate Physiological Remodelling of the Blood–Testis Barrier
Source: J Cell Physiol. 2025 Nov 18;240(11):e70109. doi: 10.1002/jcp.70109 (PMC12624519; doi:10.1002/jcp.70109)

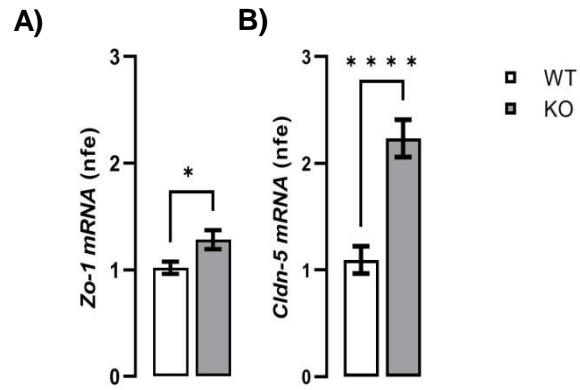

**Supplemental Figure 1**

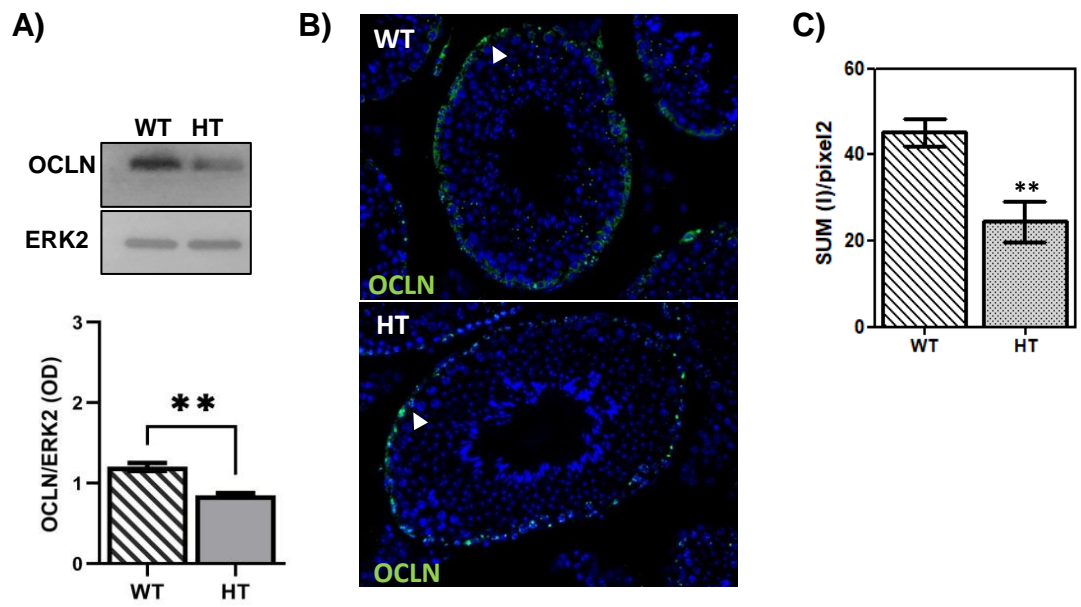

**Supplemental Figure 2**

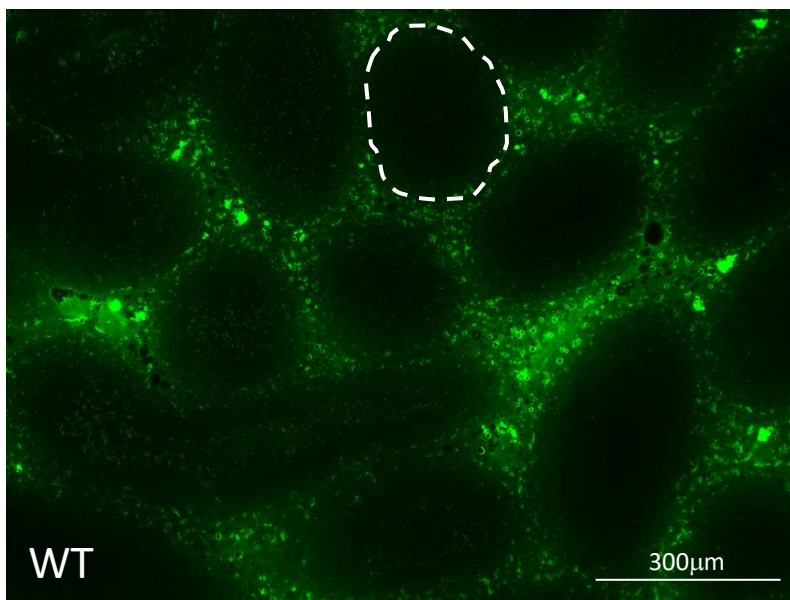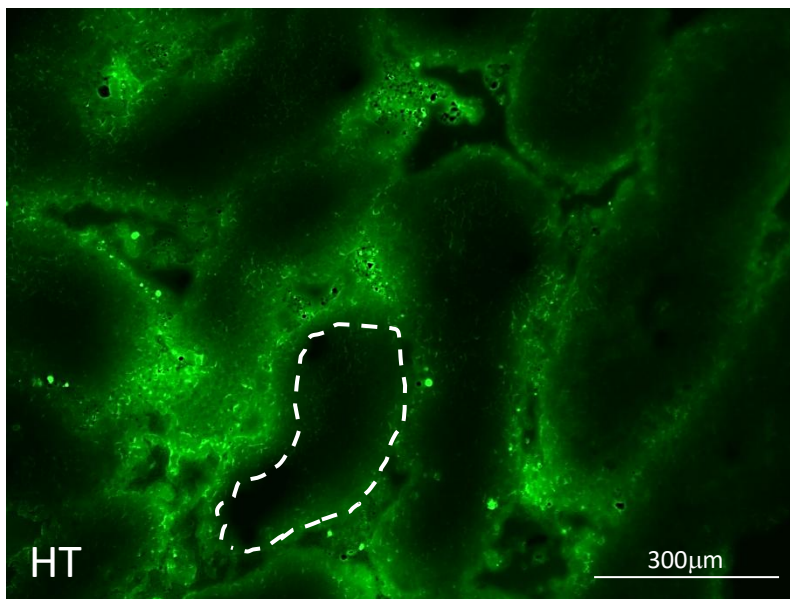

**Supplemental Figure 3**

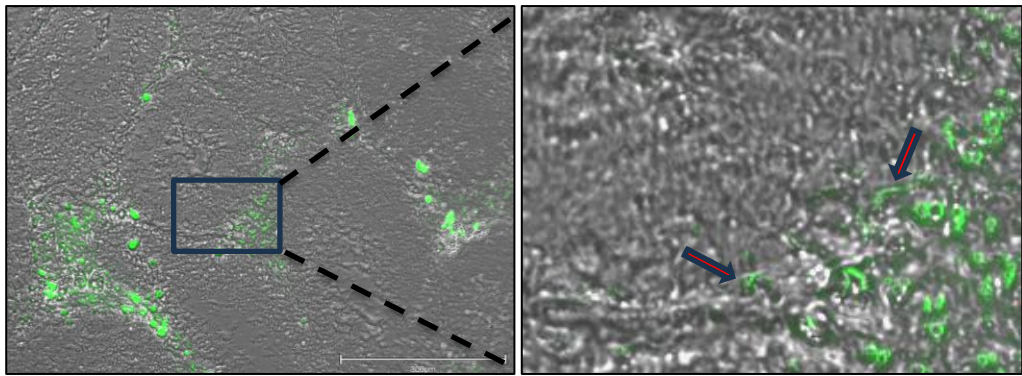

**Supplemental Figure 4**

Supplement: Supplementary file 1 — Supplementary Figures. [file JCP-240-0-s001.pdf]
